# Impact of Lipid Sources on Quality Traits of Medical Cannabis-Based Oil Preparations

**DOI:** 10.3390/molecules25132986

**Published:** 2020-06-30

**Authors:** Alberto Ramella, Gabriella Roda, Radmila Pavlovic, Michele Dei Cas, Eleonora Casagni, Giacomo Mosconi, Francisco Cecati, Paola Minghetti, Carlo Grizzetti

**Affiliations:** 1 Farmacia Dott.ri Giuliana e Alberto Ramella–SAS, Via A. Diaz 1, 21021 Angera (VA), Italy; laboratorioramella@gmail.com; 2 Department of Pharmaceutical Sciences, Università degli Studi di Milano, Via L. Mangiagalli 25, 20133 Milan, Italy; gabriella.roda@unimi.it (G.R.); eleonora.casagni@unimi.it (E.C.); paola.minghetti@unimi.it (P.M.); 3 Department of Health, Animal Science and Food Safety, University of Milan, 20133 Milan, Italy; giacomo.mosconi@unimi.it; 4 Department of Health Sciences, Università degli Studi di Milano, Via A.di Rudinì 8, 20142 Milan, Italy; michele.deicas@unimi.it; 5 INTEQUI-CONICET, Faculty of Chemistry, Biochemistry and Pharmacy, National University of San Luis, Almirante Brown 1455, San Luis CP 5700, Argentina; fmcecati@gmail.com; 6 S.S.D. Cure Palliative e Terapia del Dolore, Ospedale di Circolo–Fondazione Macchi, ASST Sette Laghi, Viale L. Borri 57, 21100 Varese, Italy; carlo.grizzetti@gmail.com

**Keywords:** cannabis oils, cannabinoids, terpenes, LCHRMS-Orbitrap, HS-SPME-GC/MS, olive oil, MCT oil

## Abstract

The feasibility of the use of two lipid sources and their impact on the cannabinoid profile, terpene fingerprint, and degradation products in medical cannabis oil preparations during 3 months of refrigerated storage time were investigated. LCHRMS-Orbitrap^®^ and HS-SPME coupled to GC-MS for the investigation of targeted and untargeted cannabinoids, terpenes, and lipid degradation products in Bedrocan^®^ and Bediol^®^ macerated oils were used as analytical approaches. As regards the cannabinoid trend during 90 days of storage, there were no differences between PhEur-grade olive oil (OOPH) and medium-chain triglycerides oil (MCT oil) coupled to a good stability of preparations for the first 60 days both in Bedrocan^®^ and Bediol^®^ oils. MCT lipid source extracted a significant concentration of terpenes compared to olive oil. Terpenes showed a different scenario since MCT oil displayed the strongest extraction capacity and conservation trend of all compounds during the shelf life. Terpenes remained stable throughout the entire storage period in MCT formulations while a significant decrease after 15 and 30 days in Bediol^®^ and Bedrocan^®^ was observed in olive oil. Therefore, MCT oil could be considered a more suitable lipid source compared to olive oil involved in the extraction of medical cannabis for magistral preparations.

## 1. Introduction

*Cannabis sativa L*. is a highly interesting officinal plant due to its efficacy for the treatment of various pathologies. The most common medical indications for its use include different diseases, such as neuropathic pain [1], glaucoma [2], undernutrition, chemotherapy-induced nausea [3,4], spasticity, and seizure in multiple sclerosis [4,5]. Cannabis chemotypes show great differences as regards their pharmaceutical properties due to close interactions, known as the ‘entourage effect’, between cannabinoids and terpenes as a result of synergic action, but scarce information is present in the literature [6]. As regards cannabinoids, great scientific effort is currently being made to investigate and characterize new compounds, evaluating their pharmacological effects [7]. Citti et al. recently described a novel cannabinoid (Δ^9^-Tetrahydrocannabiphorol) isolated from a medical cannabis chemotype with an in vivo cannabimimetic activity higher than Δ^9^-tetrahydrocannabinol (Δ^9^-THC) [8].

Medical cannabis in Italy, as in other European countries, presents an uneven scenario [9,10,11]. Dutch Bedrocan^®^ varieties (Bedrocan^®^, Bediol^®^, Bedica^®^, and Bedrolite^®^ as the most used varieties), and the new strains FM1 and FM2 produced by the Military Pharmaceutical Chemical Institute of Florence, Italy as of November 2015 by Ministerial Decree, can be prescribed to treat a wide range of diseases [12]. Medical cannabis-based prescriptions are constantly increasing in Italy, with a similar trend observed in other countries, where therapeutic use is authorized due to the positive role of cannabis in treating several pathological conditions with few side effects [13,14,15,16]. As a consequence, Italian pharmacies are legally allowed to prepare cannabis inflorescence doses for infusions, micronized capsules, vaping, and macerated oils as the most representative preparations [14,15,16,17].

Macerated oils derived from female inflorescences have received considerable attention, due to their easy dose management during the treatment period [10,11]. Cannabis oil preparations are usually prepared by using olive oil as an extraction solvent, but other lipid sources could be tested in order to define the optimal lipid source as an extraction solvent for medical cannabis-derived products [10]. Indeed, cannabidiol (CBD) oils are already prepared according to the German Cannabis Monograph and German Drug Codex (Deutscher Arzneimittel Codex, DAC) by using medium-chain triglyceride (MCT) as an extraction solvent to dissolve CBD [18,19]. Medium-chain triglycerides (MCTs) are lipids with distinctive traits as they are more readily absorbed and oxidized than most lipids [20]. This unique characteristic of MCTs has led to interest in their use to treat many diseases, including several gastrointestinal disorders, where MCTs have been primarily used to reduce fat malabsorption. In addition, MCTs represent a good source of calories, essential to manage nutritional status [20]. Some authors have investigated the gastrointestinal absorption characteristics of a drug in a lipid-containing oral dosage form in rats in relation to the digestibility of lipids, confirming MCTs as an efficient vehicle leading to better bioavailability compared to long-chain triglycerides (LCTs) [21,22].

Moreover, in recent literature on medical cannabis preparations and chemotypes, many papers investigated different extraction parameters and their influence on the final derived products as well [9,10,11,13,14,15,16,17]. In particular, the decarboxylation time and temperature, maceration conditions, and cannabinoid and terpene trends during storage have been studied to select the best conditions able to preserve the phytocomplex of the original cannabis plant composition [17].

On the other hand, much research has focused attention on the development of analytical methods based on liquid chromatography coupled to UV detection (HPLC-UV) together with mass spectrometry (LC-MS/MS and HRMS) to quantify targeted and untargeted cannabinoids, terpenes, and oxidation products, such as aldehydes and ketones, both in inflorescences and derived oils at the ppb level [9,10,11,23,24,25,26,27,28] as summarized in Table 1.

However, no evidence is available in the literature detailing losses during all preparation phases, including the maceration of inflorescences into oil and final filtration, required to obtain pharmaceutical oils. The in-depth investigation of losses occurring during the different preparation phases, in particular focused on decarboxylation, maceration, and filtration, is crucial to evaluate process efficiency, which has, until now, been presented only in terms of the active compound concentrations obtained in final oils. These aspects are strategic in order to transfer a standardized protocol to authorized pharmacies involved in medical cannabis product formulation. In our previous work, following a detailed morphological survey of plant material, the effect of extraction conditions on oily preparations and temperature on cannabinoid decarboxylation in the cannabis inflorescence was evaluated [11]. To harmonize extraction methods and temperatures, the Italian Society of Compounding Pharmacists (SIFAP) recently proposed a method of preparation to pharmacists. The concentration of cannabinoids was studied to optimize extraction, thus reducing variability among preparations [11].

Based on the above considerations, this research aimed to investigate the feasibility of the use of two lipid sources, PhEur-grade olive oil (OOPH) and MCT oil, and their impact on the principal cannabinoid profile (quantity of Δ^9^-THC, Δ^9^-tetrahydrocannabinoic acid (Δ^9^-THCA), CBD, cannabidiolic acid (CBDA), and cannabinol (CBN), Figure S1), terpene fingerprint, and oxidation products in two medical-based cannabis oil preparations over 3 months of refrigerated storage time. Bedrocan^®^ and Bediol^®^ medical cannabis chemotypes are involved in the present study since they possess different proportions of cannabinoids (Bedrocan^®^ 22% THC, <1% CBD; Bediol^®^ 6.5% THC, 8% CBD). In addition, these inflorescences are the most common varieties actually prescribed for disease treatment. The entire preparation method used to obtain the medical cannabis oils was also evaluated throughout to assess its efficiency so it could be transmitted to pharmacies as a standardized preparation protocol.

## 2. Results and Discussion

### 2.1. Medical Cannabis-Based Oil Extraction Procedure

The preparation steps to obtain cannabis-based oils are detailed in Table 2. As regards the two different chemotype and lipid sources involved in this study, no significant differences were observed between oils. High extraction efficiency was obtained as a result of standardized steps that are critical to extract plant material by using oil as a solvent, resulting in minimal plant parts remaining at the end of the entire procedure. The high extraction efficiency observed in this study is probably also due to the last step of the preparation protocol, which is essential to extract all oil from residual plant inflorescences. Comparing the results of this research with the literature, no study to date has investigated losses throughout the entire oil preparation protocol, calculating the final extraction yield [11]. This is crucial to optimize every single working step in order to minimize cannabis waste as well as being particularly important for pharmacies involved in the preparation of cannabis products. Most of the published methods do not report the use of any mechanical pressure to extract the final oil, thus leading to oil losses remaining in waste cannabis inflorescences used for magistral preparations. This could have a great impact on the extraction efficiency as well as on the subsequent active compound concentrations of cannabinoids and terpenes.

### 2.2. Fatty Acid Composition of Lipid Sources

**Figure 1:**
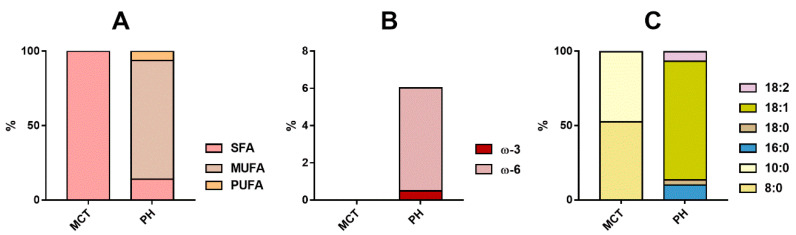
Fatty acid composition PhEur-grade olive oil (OOPH) and medium-chain triglyceride (MCT) oils. Comparison between the composition (**A**) in saturated (SFA) and unsaturated fatty acids (monounsaturated fatty acids, MUFAs; polyunsaturated fatty acids, PUFAs); (**B**) in omega-3 and omega-6 polyunsaturated fatty acids (**C**) in the main species of fatty acids. Data are expressed as percentage considering the mass of the individual fatty acids.

Fatty acid composition was determined to evaluate the different compositions of lipid sources used for preparations. Figure 1 shows the analysis of the lipidic component of OOPH and MCT oils used for the preparation of cannabis oleolites. OOPH oil was composed mainly of monounsaturated fatty acids (MUFAs), such as C18:1 (78.8%) and C18:2 (5.2%) acids, along with saturated fatty acids (SFAs) C18:0 (3.4%) and C16:0 (10.6%) fatty acids. On the other hand, MCT oil showed only medium-chain saturated fatty acids, in particular C8:0 (52.4%) and C10:0 (47.2%). MCT oil is composed of triglycerides containing short- and medium-chain saturated fatty acids, in particular C6:0, C8:0, C10:0, and C12:0. On the contrary, OOPH consisted mainly in a mixture of saturated and unsaturated long-chain fatty acids. Specifications from Pharmacopoeia reported the following composition in fatty acids: C16:0 (7.5–20%), C18:0 (0.5–5%), C18:1 (56–85%), C18:2 (3.5–20%), and others [29].

In general, lipid sources rich in saturated fatty acids are less sensitive to oxidation phenomena after hot maceration, leading to minimal deterioration during the subsequent storage as shown in our previous research [17]. The critical factors influencing the oxidation stability of lipid-based products are the temperature during preparation, storage, and the lipid used [17]. No previous information was available investigating different lipids as solvents for cannabis extraction. In 2013, Romano and Hazekamp [26] evaluated solvents, such as naphtha, petroleum ether, ethanol, and olive oil, as they possess different polarity and extraction efficiency as regards cannabinoids from cannabis. Considering their final use as therapeutic products and for human consumption, solvents, such as naphtha and petroleum, must be avoided due to their toxic impact on human health. In addition, alcohol-based methods used to extract active compounds from cannabis inflorescences to obtain concentrated extracts are at present not recognized and allowed by the Italian Ministry of Heath nor by other European countries [12].

In addition, other considerations must be highlighted concerning MCT lipid used for drug development or taken as a dietary supplement. MCTs differ from long-chain triglycerides (LCTs) in several physicochemical characteristics, such as their smaller molecular size. MCTs are hydrolyzed both faster and more extensively during digestion [30]. Most of the remaining non-hydrolyzed MCTs are readily absorbed by intestinal cells. In addition, medium-chain fatty acids MCFAs show greater solubility in aqueous media while remaining capable of passive, non-rate-limiting diffusion across cell membranes because of their relatively short chains. MCFAs show a low affinity for anabolic enzymes (such as diglyceride acyltransferase), therefore undergoing minimal re-esterification, a process necessary for de novo synthesis of triglycerides (TGs) [30]. Once absorbed during digestion, most MCFAs and MCTs are transported through the portal system directly to the liver with minimal mobilization of chylomicrons while long-chain fatty acids (LCFAs) are packed in chylomicrons prior to their shipment to the periphery mainly via the lymphatic system [31]. The easy absorption of MCTs without the need for bile or pancreatic enzymes makes them a good source of calories in the setting of malabsorption and steatorrhea from diseases, such as pancreatic or bile insufficiency [31]. MCT-based lipids are already utilized in different pathologies, such as chronic intestinal inflammation and other similar illnesses. In addition, several studies had focused attention on the bioavailability of the oral drug diazepam [21] or natural compounds, such as vitamin E [32] and quercetin [33], prepared by using MCT lipid, confirming increased intestinal adsorption and plasma concentration as a result of the better efficiency of MCTs. This could be interesting considering the applications of cannabis oil preparations since many patients treated with cannabis are affected by more than one pathology, including intestinal inflammation and chemotherapy-induced anorexia besides chronic pain. Another crucial aspect related to the greater bioavailability of MCTs could be represented by the modification of the pharmacokinetic traits of cannabis oil.

### 2.3. Targeted Cannabinoids in Pharmacists’ Oil Preparations

The five main cannabinoids were quantified according to the recently published HPLC-Q-Exactive-Orbitrap-MS method [17]. All cannabinoid amounts (ppm) quantified in different preparations during storage are presented in Tables S1 and S2. Coefficients of variation (CV%) in the concentration of cannabinoids at T0 in Bediol^®^ and Bedrocan^®^ oils between the lipid sources used are presented in Table 3.

**Figure 2:**
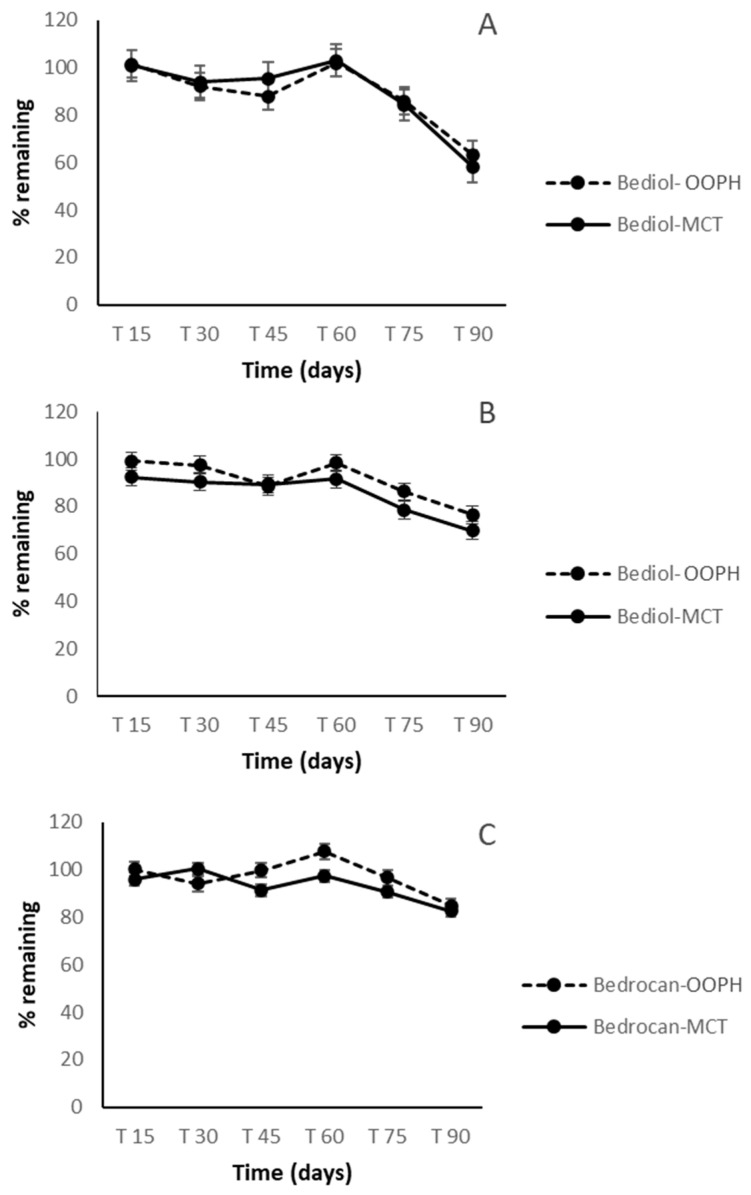
Δ^9^-THC trends in Bediol^®^ (**A**), Bedrocan^®^ (**C**), and CBD in Bediol^®^ (**B**)-based oils as a function of the storage time and lipid sources: PhEur-grade olive oil (OOPH) and medium-chain triglycerides (MCTs).

The concentration of Δ^9^-THC in Bedrocan^®^ preparations was about 1.9–2.0% (*w*/*w*). The concentration of Δ^9^-THC in Bediol^®^ preparations was around 0.7–0.8% (*w*/*w*), while that of CBD was about 0.8–0.9% (*w*/*w*) and no significant differences were observed between OOPH and MCT oils for both cannabis varieties. As regards cannabinoid trends during 90 days of storage, there were no significant differences between OOPH and MCT oils together with a good stability of preparations for the first 60 days both in Bedrocan^®^ and Bediol^®^ oils (Figure 2). In addition, the two lipid sources were similar regarding cannabinoid concentration (CV% < 15%, Table 3).

**Figure 3:**
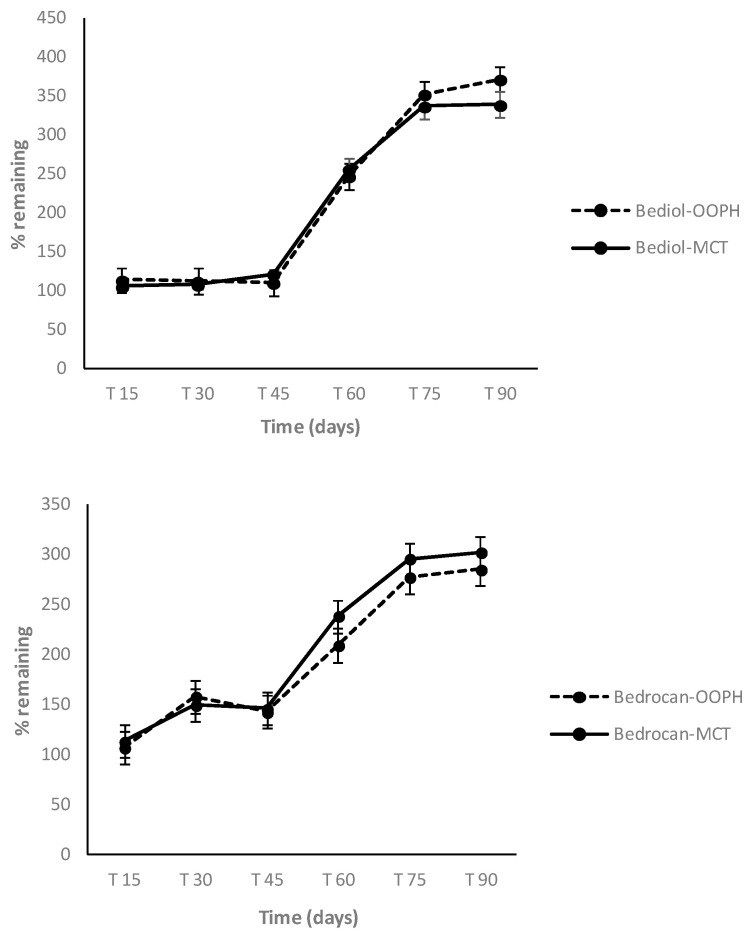
CBN (cannabinol) trends in Bediol^®^ and Bedrocan^®^-based oils as a function of storage time and lipid sources: PhEur-grade olive oil (OOPH) and medium-chain triglycerides (MCTs).

**Figure 4:**
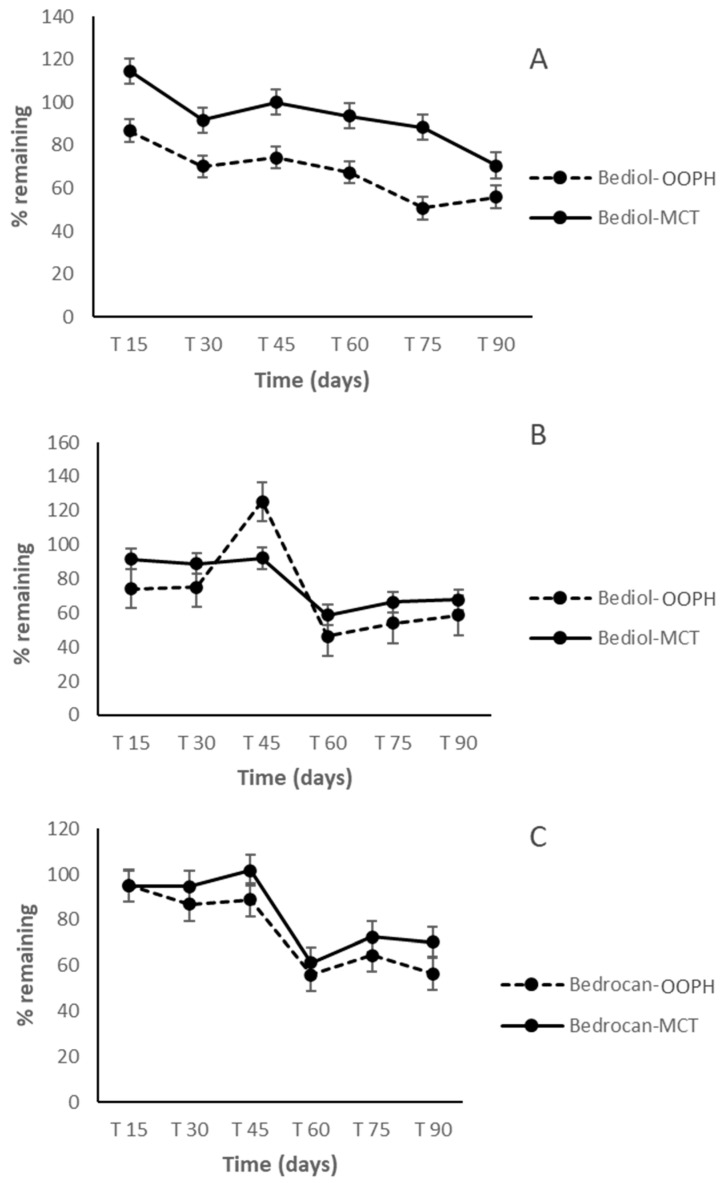
CBDA (cannabidiolic acid) trends in Bediol^®^ (**A**), Δ^9^-THCA in Bediol^®^ (**B**) and Bedrocan^®^ (**C**)-based oils as a function of storage time and lipid sources: PhEur-grade olive oil (OOPH) and medium-chain triglycerides (MCTs).

In the following period, from 60 to 90 days, Δ^9^-THC and CBD were more stable, in both oils and in both cannabis varieties, and their concentration started to decrease after 75 days (*p* < 0.05). On the other hand, Δ^9^-THCA and CBDA showed a marked concentration decrease after just 60 days. An increase of CBN was registered after 45 days, due to the contemporary oxidation of Δ^9^-THC (Figure 3 and Figure 4).

In contrast to previous studies on oil preparation [14,15,16,17], which showed that the greatest decline in cannabinoid content occurred during the first week of storage, the oil preparations in both mediums examined in this study were exceptionally stable up to 60 days regarding the two major components, Δ^9^-THC and CBD. This indicates that the extraction procedure applied herein was effective not only as regards the qualitative cannabinoid profile but also regarding the stability of preparations during the period of consumption. The higher variability noted for acidic forms, particularly Δ^9^-THCA in the Bediol^®^ olive oil preparation at 45 days of storage time, postulates a possible interaction between acidic cannabinoids and long-chain monounsaturated fatty acids from OOPH oil. In addition, the increase of some similar compounds observed in this research was probably related to the non-homogeneous distribution of some plant residues, even if minimal, that could remain in the oil [17].

### 2.4. Untargeted Cannabinoids in Pharmacists’ Oil Preparations

**Figure 5:**
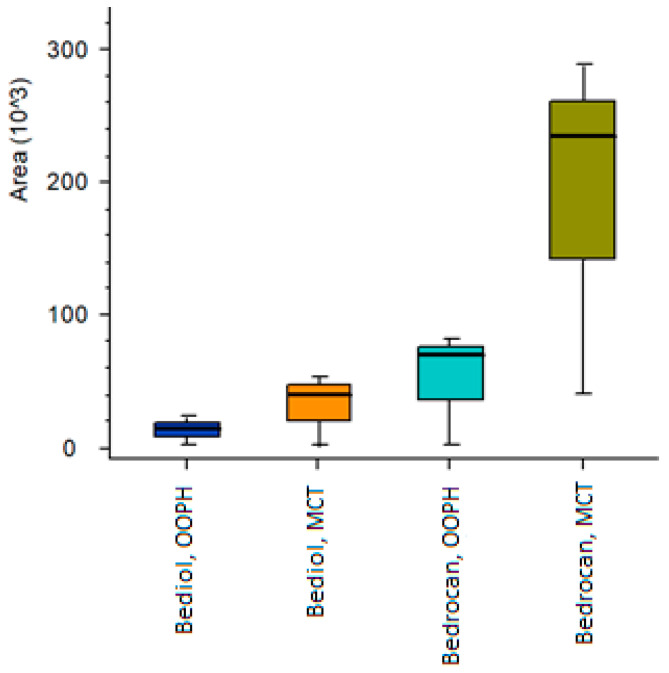
Δ^9^-Tetrahydrocannabiphorol (Δ^9^-THCP) relative amount expressed as the normalized peak area in investigated oleates. Data are reported as median with 25th–75th percentile range on three biological repetitions.

Q-Exactive-Orbitrap-high-resolution mass spectrometry (HRMS) offers the opportunity to perform “in-depth” cannabinoid profiling in magistral oil preparations as it uniquely provides accurate molecular masses and specific fragmentation patterns for detected species [9,10,34,35]. Moreover, HRMS acquisition mode accumulates all sample data, enabling the identification of “unpredicted” compounds with a cannabinolic structure and retrospective data analysis without the need to re-run samples. This detection technique has proven to be particularly reliable, as there is no risk of native cannabinoid decomposition (decarboxylation of cannabinoid acids during analysis), which may compromise the accurate assessment of the overall cannabinoid profile [17,34]. For this reason, the freshly prepared Bedrocan^®^ and Bediol^®^ magistral oil samples were subjected to untargeted cannabinoid investigation. This analytical approach revealed the presence of minor cannabinoids belonging to those already previously classed [9,10,34,35,36]. The analytical platform that includes the elaboration of data by Compound Discoverer software revealed the presence of different Δ^9^-THC and CBD analogues that contained C3 (*m*/*z* = 287.2006) and C4 (*m*/*z* = 301.2162) side chains instead of C5 [34,35]. These compounds were also followed by traces of their acidic forms. Moreover, cannabichromen (CBC) (CBD and Δ^9^-THC isomer with a longer retention time) and its acidic form were particularly emphasized in the Bedrocan^®^ preparation regardless of the oil type. Bediol^®^ preparations revealed the presence of cannabielsoin (CBE) (*m*/*z* = 331.2268) that is formed by oxidative modifications of CBD. All samples contained cannabigerolic acid (CBGA, *m*/*z* = 361.2375) and its neutral form (CBG, *m*/*z* = 317.2475). Furthermore, attention was paid to the presence of a recently discovered novel cannabinoid, namely Δ^9^-Tetrahydrocannabiphorol (Δ^9^-THCP) [8]. The signal that corresponds to its pseudomolecular cation (C_23_H_35_O_2_^+^ theorical mass 343.26316; found: 343.26274) accompanied with two fragments (221.15390 and 287.20089) emerged in all oil preparations. Nevertheless, the relative amount (normalized peak area) of Δ^9^-THCP in both MCT oleates (Bedrocan^®^ and Bediol^®^) was higher than in the corresponding olive oil-based preparations (Figure 5). This finding leads to the hypothesis that Δ^9^-THCP as a seven-termed side chain cannabinoid that possesses pronounced lipophilicity, which is why its extraction yield is higher with MCT medium. At the same time, relatively higher amounts found in Bedrocan^®^-MCT deserve further study as it was demonstrated that this heptyl Δ^9^-THC homologous showed a cannabimimetic activity several times higher than Δ^9^-THC, both under in vitro and in vivo conditions [8]. The discovery of this extremely potent Δ^9^-THC-like phytocannabinoid in both the Bedrocan^®^ and Bediol^®^ chemotype may explain several pharmacological effects not attributable exclusively to Δ^9^-THC [37]. The results of this study, although preliminary, indicate that in deep investigation toward the presence of untarget cannabonoid, such as Δ^9^-THCP, would be strategic, in order to characterize the different cannabis chemotypes involved in disease treatment.

### 2.5. Terpene Profile in Pharmacists’ Oil Preparations

**Figure 6:**
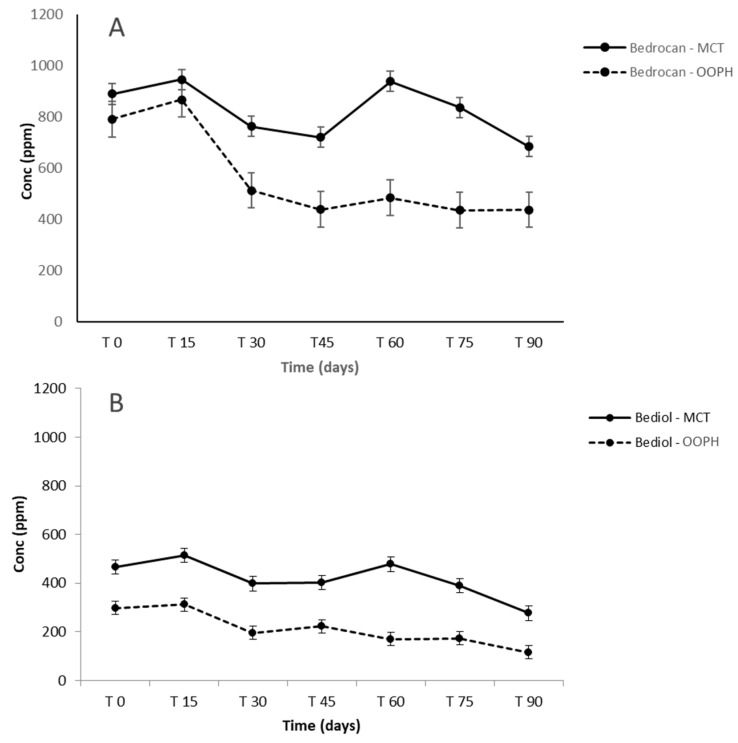
Concentration of the total extracted amount of terpenes (ppm) in Bedrocan^®^ (**A**) and Bediol^®^ (**B**)-based oils as a function of the storage time and lipid sources: PhEur-grade olive oil (OOPH) and medium-chain triglycerides (MCTs).

**Figure 7:**
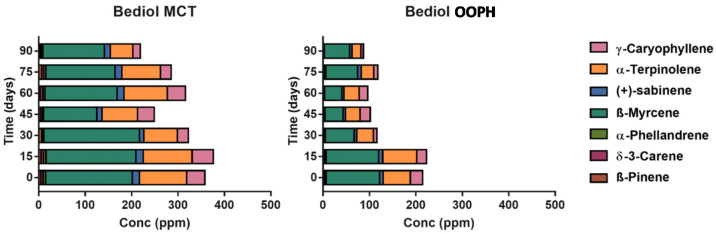
Concentration of the most representative terpene species (ppm) in Bediol^®^ oils as a function of storage time and lipid sources: PhEur-grade olive oil (OOPH) and medium-chain triglyceride (MCT) oils.

**Figure 8:**
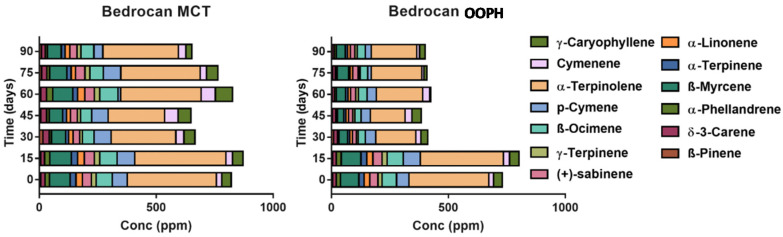
Concentration of the most representative terpene species (ppm) in Bedrocan^®^ oils as a function of the storage time and lipid: PhEur-grade olive oil (OOPH) and medium-chain triglyceride (MCT) oils.

The overall terpenes and other volatile compounds (VOCs) classified according to their chemical classes and their volatile compounds are presented in Tables S3–S6, while concentration of the total extracted amount of terpenes was shown in Figure 6. Different terpene profiles characterizing Bedrocan^®^ (chemotype I) and Bediol^®^ (chemotype II) were observed as also presented in Figure 7 and Figure 8 focused on the most abundant compounds [38].

A different scenario was shown in the terpene profile since MCT oil displays a stronger extraction capacity and a better tendency to preserve all compounds during shelf life (Figure 6, Tables S3–S6). After 90 days, a decrease in MCT as regards T0 was shown in terpene levels of about −40.70% (Bediol^®^) and −23.07% (Bedrocan^®^), with −61.27% (Bediol^®^) and −44.70% (Bedrocan^®^) in OOPH olive oil. Moreover, MCT oil demonstrated a better terpene extraction capacity, measured at T0, than PH olive oil: +56.63% in Bediol^®^ and +12.35% in Bedrocan^®^ (MCT vs. OOPH). The increase of the concentration in some terpenes during storage had already been observed in our previous research, probably due to the non-homogeneous distribution of plant residues, even if minimal, that could remain in the oil [17]. To conclude, MCT oil appears to be a more suitable lipid source than OOPH in maintaining the phytochemical traits of cannabis inflorescences used for magistral preparations. This is important since terpenes act together with cannabinoids through the well-documented “entourage effect” [6], leading to the pharmacological-active properties of magistral oil. As a last consideration, aldehydes and in particular hexanal arising from the oxidation of linoleic acid are present in lower amounts in MCT-based oils, confirming that the role of lipids is strategic in conferring greater stability during storage as shown in our previous research [17,39].

In Figure 7 and Figure 8, the most abundant terpenes were presented in the two different cannabis-based oils. The better stability of terpenes extracted by using MCT lipid during storage was confirmed after examining the rapid decrease after 15 days (*p* < 0.05). This phenomenon is probably related to the greater stability of the entire phytocomplex against oxidation phenomena, which leads to rapid terpene losses, since saturated lipids are more stable as regards oxidation. Comparing these results with our previous research focalized on investigating the role of thermal treatment during oil preparation conducted with Bedrocan^®^ and Beldiol^®^ chemotypes, a similar trend was observed regarding the extraction based on the ultrasound process [17].

## 3. Materials and Methods

### 3.1. Chemicals and Reagents

All HPLC analytical-grade solvents and chemicals were purchased from Sigma (Sigma–Aldrich, St. Louis, MO, USA). Formic acid 98–100% was from Fluka (Sigma–Aldrich, St. Louis, MO, USA). Ultrapure water was obtained through a Milli-Q system (Millipore, Merck KGaA, Darmstadt, Germany). For HS analysis, the solid phase microextraction (SPME) coating fiber (divinylbenzene-carbowax-polydimethylsiloxane, DVB/CAR/PDMS, 50/30 µm) was from Supelco (Bellefonte, PA, USA). Olive oil was purchased from Galeno (Carmignao, PO, IT) and MCT oil from Farmalabor (Canosa di Puglia, BT, IT). All cannabinoids were analytical standards at a concentration of 1.0 mg/mL in methanol, ampule of 1 mL, certified reference material, Cerilliant^®^, Sigma Aldrich, Round Rock, Texas. The standard mixtures of 37 FAMEs (FAME Mix, SUPELCO, Bellefonte, PA, USA), 0.2 mg/mL and 0.4 mg/mL in methylene chloride (analytical standards), and methyl-undecanoate, certified reference material, TraceCERT^®^, (internal standard) were purchased from Sigma Aldrich Chemical Co. (St. Louis, MO, USA).

### 3.2. Medical Cannabis-Based Oil Preparation Procedure

Bedrocan^®^ and Bediol^®^ medical cannabis varieties were used in the present study as they are characterized by different proportions of major cannabinoids (Bedrocan^®^: 22% Δ^9^-THC, <1% CBD; Bediol^®^ 6.5% Δ^9^-THC, 8% CBD) and are also widely used in therapeutic protocols to manage many disease conditions, including chronic pain and chemotherapy side effects as well as many others.

All experimental conditions adopted are summarized in Table 4. Regarding the extraction method, preliminary experiments were previously conducted in order to select some method parameters to help to standardize the final magistral preparations [11]. In particular, the impact of the temperature and extraction time on the concentration of cannabinoids was investigated to optimize extraction so as to reduce variability among preparations [11]. These parameters are crucial since they are closely related to the conversion of cannabinoids from their acid to their neutral form and are also able to modify the terpenes present in final magistral oils [17]. Based on the optimal decarboxylation and extraction conditions, 125 °C for 30 min and 100 °C for 30 min, respectively, were used in this study.

All samples of magistral oils were finally transferred into 50-mL amber glass bottles to protect the oil from light and were then stored at 4 °C. The refrigerated storage temperature was selected based on recent research in which a comparison of storage temperatures was performed confirming room temperature as leading to a greater active compound decrease during storage compared to 4 °C [17]. The bottles were shaken and opened twice a day to simulate real use conditions. All analyses were performed at 0, 15, 30, 45, 60, 75, and 90 days of storage. According to the best available literature, 90 days of storage had not been tested to date (Tables S1 and S2).

### 3.3. Cannabinoid HPLC-Q-Exactive-Orbitrap-MS Analysis

The content of five major cannabinoids was assessed by a recently published validated analytical method [9,10,17]. Briefly, the oil samples were prepared by dissolving 100 mg of each oil in 10 mL of isopropanol. After adding 1 µg/mL of IS, 10 µL of each sample were diluted in 890 µL of initial mobile phase from which 2 µL were injected.

#### 3.3.1. Targeted Approach by Quantitative Analysis

Chromatographic analysis was performed on HPLC system (Thermo Fisher Scientific, San Jose, CA, USA) that consisted of a Surveyor MS quaternary pump with a degasser, a Surveyor AS autosampler with a column oven, and a Rheodyne valve with a 20-μL loop. A reverse-phase HPLC column 150 × 2 mm i.d., 4 μm, Synergi Hydro RP, with a 4 × 3 mm i.d. C18 guard column (Phenomenex, Torrance, CA, USA) was used for separation. The column oven and autosampler temperatures were 30 and 5 °C, respectively. The mobile phase was made up of 0.1% aqueous formic acid and acetonitrile with 0.1% formic acid. The gradient (0.3 mL/min) was started with 50% of aqueous eluent with a linear decrease up to 5% in 50 min, which was maintained for 2 min. The mobile phase was returned to initial conditions in the next two minutes, and the rest of the run (total time: 30 min) was used for re-equilibration. The mass spectrometer Thermo Q-Exactive Plus (Thermo Scientific, San Jose, CA, USA) with a HESI source had a capillary temperature and vaporizer temperature set at 330 and 280 °C, respectively. The electrospray voltage operating in positive was adjusted at 3.50 kV while the sheath and auxiliary gas were 35 and 15 arbitrary units, with an S lens RF level of 60. The mass spectrometer was under control of the Xcalibur 3.0 software (Thermo Fisher Scientific, San Jose, CA, USA). The FS (full scan acquisition) was adjusted on 70,000 FWHM at *m*/*z* 200, with a scan range of *m*/*z* 215–500. The automatic gain control (AGC) was set at 1e^6^, with an injection time of 100 ms. Detection was based on the calculated exact mass of the protonated molecular ions, isotopic pattern, and retention time of the target compounds [12]. Extracted ion chromatograms (EICs) were obtained with an accuracy of 2 ppm *m*/*z* from the total ion chromatogram (TIC) engaging the *m*/*z* corresponding to the molecular ions [M + H]^+^ 315,23145 for CBD and Δ^9^-THC, 311,20020 for CBN, 317.24716 for CBG, and 311.2024 for CBN. Retention times were as follows: 16.5 min (CBDA), 17.3 min CBD, 19.8 min (CBN), and 21.2 min Δ^9^-THC and 23.1 (Δ^9^-THCA).

#### 3.3.2. Untargeted Approach by Investigating for Novel Cannabinoids

HPLC conditions were the same as those indicated above for targeted analysis (the mobile phase consisted of water and acetonitrile gradient both acidified with 0.1% formic acid) but with a gradient that started with 95% of 0.1% aqueous formic acid with a linear decrease to 5% in 30 min. The chromatographic run was returned to initial conditions at 35 min, followed by a 5-min re-equilibration period. The untargeted MS/MS (dd-MS^2^) analysis operated the resolution at 35,000 FWHM (*m*/*z* 200). The AGC target was set to 2e^5^, with the maximum injection time of 100 ms. The fragmentation of precursors was optimized as two-stepped normalized collision energy (NCE) (25 and 40 eV). The FS-dd-MS^2^ (full scan data-dependent acquisition) was used for both screening and quantification purposes. The resolving power of FS was adjusted on 140,000 FWHM at *m*/*z* 200, with a scan range of *m*/*z* 215–500. The automatic gain control (AGC) was set at 3e^6^, with an injection time of 200 ms. A targeted MS/MS (dd-MS^2^) analysis operated at a resolution of 35,000 FWHM (*m*/*z* 200). The AGC target was set to 2e^5^, with the maximum injection time of 100 ms. Fragmentation of precursors was optimized as two-stepped normalized collision energy (NCE) (25 and 40 eV). Detection was based on the calculated exact mass of the protonated molecular ions, at least one corresponding fragment, and eventually on the retention time of detected compounds by applying the identification strategy explained recently [35,36].

### 3.4. HS-SPME and GC-MS Analysis for Terpene Analysis

To evaluate the volatile profile (VOCs) and terpenes as a primary focus, headspace analysis by using solid-phase microextraction was used since it represents the most suitable technique to investigate the terpene profile characteristics of several officinal plants [40,41,42]. All method parameters of the fiber type, time, and extraction temperature have already been investigated in our published studies on medical cannabis chemotypes and hemp variety inflorescences [40,41,42]. Briefly, 100 mg of each oil sample were placed into 20-mL glass vials along with 100 μL of the IS (4-metil-2-pentanone, 20 µg/mL in 2-propanol). A silicon/PTFE septum-based cap (Supelco, Bellefonte, PA, USA) was used to close the vial, which was then kept in the temperature block (37 °C) (CTC Analytics, Zwingen, Switzerland). At the end of the sample equilibration time (30 min), a conditioned (60 min at 280 °C) SPME fiber was subjected to the sample for 120 min using a CombiPAL system injector autosampler (CTC Analytics, Zwingen, Switzerland). After sampling, the SPME fiber was immediately inserted into the GC injector and thermally desorbed. A desorption time of 1 min at 230 °C was used in the splitless mode. Before sampling, each fiber was reconditioned for 5 min in the GC injector port at 230 °C.

GC-MS analyses were performed with a Trace GC Ultra coupled to a Trace DSQII quadrupole mass spectrometer (MS) (Thermo-Fisher Scientific, Waltham, MA, USA) equipped with an Rtx-Wax column (30 m × 0.25 mm i.d. × 0.25 µm film thickness) (Restek, Bellefonte, PA, USA). The oven temperature program was: from 35 °C, held for 8 min, to 60 °C at 4 °C/min, then from 60 to 160 °C at 6 °C/min, and finally from 160 to 200 at 20 °C/min. Helium was the carrier gas, at a flow rate of 1 mL/min. The MS was operated in electron impact (EI) ionization mode at 70 eV. Mass spectra were obtained by using a mass selective detector, a multiplier voltage of 1456 V, and by collecting the data at rate of 1 scan/s over the *m*/*z* range of 35–350. An alkane mixture (C8–C22, Sigma R 8769, Saint Louis, MO, USA) was injected under the same chromatographic conditions to obtain the Kovats retention indices (RI) for each detected compound [41,42,43]. The identification of compounds was conducted by comparing the retention times of the chromatographic peaks with those of authentic compounds analyzed under the same conditions when available, or by comparing the Kovats retention indices with literature data and through the National Institute of Standards and Technology (NIST) MS spectral database. The results were finally expressed as µg/g IS equivalents.

### 3.5. Fatty Acid Composition of Lipid Sources

Evaluation of the fatty acid profile characterizing the two lipids involved in this research was conducted according to Orsavova et al. 2015 [43]. Briefly, 4 mL of 0.5 M sodium hydroxide in methanol were added to 1 mL of oil sample, and heated for 20 min. Furthermore, 5 mL of 15% boron trifluoride in methanol were added to methylate the samples. Then, 5 mL of hexane and 2 mL of saturated solution of sodium chloride were added and the sample was removed from the heating block (CTC Analytics, Zwingen, Switzerland). Next, 15 mL of hexane and 40 mL of saturated solution of sodium chloride were added to the extract of FAMEs, the mixture was shaken, and phases were separated and washed subsequently with 40 mL of saturated solution of sodium chloride. The organic phase was separated, and anhydrous sodium sulphate was added. Quantitative determinations of FAMEs were conducted according to [44] using a Thermo Trace Ultra gas chromatograph (Thermo-Fisher Scientific, Waltham, MA, USA) with a flame ionization detector (FID) and capillary column Restek 2560 (100 m × 0.25 mm) with a stationary phase (88% cyanopropyl, aryl-polysiloxan) with the thickness of 0.2 μm. Then, 1 mL of methyl-undecanoate was used as the internal standard. The injection volume was 1.0 μL, nitrogen was used as a carrier gas, and the detector temperature was set at 260 °C. The oven program temperature was 70 °C/5 min, 200 °C/30 min, and 240 °C/15 min. The injection temperature was 250 °C with the split ratio of 1:50. Identification of FAMEs was performed by comparing their retention times with those of reference standards (mixture FAME Mix, SUPELCO, which included 37 FAMEs). For the quantification of FAMEs, methyl-undecanoate (Sigma Aldrich Chemical Co., St. Louis, MO, USA) was used as the internal standard. The results of FAs were expressed as percentages of total fatty acids considering the mass of the individual fatty acids.

### 3.6. Data Analysis

The stability for all investigated formulations was defined as the time at which 90% of the initial concentration of target cannabinoid remained (t90); the initial concentration (time point 0) was considered to be 100%. The active compound concentration was finally expressed as the percentage of the initial active compound concentration remaining at each sampling time. Statistical evaluation of the data was conducted by using SPSS software, Version 24; Chicago, IL: SPSS Inc; 2002. Analysis of variance (ANOVA) was applied to the data and significant differences were determined by one-way ANOVA and Student–Newman–Keuls (SNK) as a post-hoc test to evaluate differences among different formulations and storage times. An effect was considered significant at a 5% level (*p* < 0.05).

Additionally, for the untargeted compounds, raw data from Xcalibur 3.0 software were processed with the Compound Discoverer™ platform (Thermo Scientific, Waltham, MA, USA) that enables peak detection, retention time adjustment, profile assignment, and isotope annotation. The relative intensity of the Δ^9^-THCP chromatographic peak from two oil types was processed by Compound Discoverer™ software that automatically displays the distribution for each compound as a box-and-whiskers plot.

## 4. Conclusions

As cannabis extracts are being used ever more frequently, the investigation of two lipid sources for the extraction of cannabinoids and terpenes is extremely important. This study demonstrated that the MCT lipid source could represent a valid lipid to be used for the formulation of medical cannabis-based oils. The terpenes extracted by using MCT in Bediol and Bedrocan chemotypes were more abundant than in olive oil. Considering the main representative terpenes, significant losses were observed after 15 and 30 days of storage in Bediol and Bedrocan, respectively. Moreover, terpenes are today considered an active compound class for their documented entourage effect and MCT was able to preserve these compounds during 90 days of storage. In addition, the preparation using MCT was richer in the novel cannabinoid characterized by a pharmacological activity, confirming this lipid source as more appropriate to extract active and functional compounds from cannabis inflorescences. Furthermore, the extraction method described could be proposed as a practical guide for pharmacists as it provides high extraction efficiency and a good amount of decarboxylated Δ^9^-THC and CBD.

## Figures and Tables

**Table 1:** Research investigation on cannabis-based oily preparations.

Author	Year	Cannabis Oil Typology	Determination	Stability Tests	Analytical Method
**Pavlovic et al.** [9]	2018	Cannabidiol Oils, European Commercially Available Preparations, Bedrolite^®^ oil extract	Cannabinoids Content, Terpene Fingerprint, Secondary Lipid’s Oxidation Products	No	SPME-GC-MS and HPLC-Q-Exactive-Orbitrap-MS
**Pacifici et al.** [14]	2017	Standardized preparations of cannabis oil FM2	Cannabinoids concentration	Yes, short-term (up to 14 days) stability and after 1 year of storage in darkness at 4 °C	UHPLC-MS/MS
**Pacifici et al.** [15]	2018	Standardized preparations of cannabis oil FM2	Cannabinoids concentration, Cannabinoids extraction efficiency	Yes, samples stored at room temperature (25 °C) and in the refrigerator (4 °C) at the following timeintervals: 1, 3, 7, and 14 days.	UHPLC-MS/MS
**Citti et al.** [16]	2016	Cannabis-based extracts–different solvent (Olive oil and ethyl alcohol)	Cannabinoids concentration and their stability, Terpenes	Short-term stability in olive oil and ethyl alcohol for 24 h at room temperature and at 10 °C. Stability of CBDA, CBD, CBN, Δ^9^-THC and THCA at room temperature (25 °C) and in a refrigerator (8 °C) for 3, 5, 6, and 10 days.	HPLC-UV, HPLC-ESI-QTOF, GC–MS
**Calvi et al.** [17]	2017	Macerated oils, Bedrocan^®^, Bediol^®^	In-depth fingerprint of volatile compounds, investigation of targeted and untargeted cannabinoids	Yes, storage at 4 and 25 °C for 6 weeks, the analyses were performed at 0, 7, 14, 21, 28, 35, and 42 days of storage.	HS-SPME coupled to GC–MS and LC-HRMS (q-exactive orbitrap^®^)
**Deidda et al.** [25]	2019	Cannabis olive oil extracts	Cannabinoids (CBD and Δ^9^-THC)	No	RP-HPLC/UV
**Romano et al.** [26]	2013	Concentrated cannabis extracts, Rick Simpson oil, Bedrocan^®^	Cannabinoids, terpenes	No	GC/FID, HPLC/PDA
**Bettiol et al.** [27]	2019	magistral oil preparations, Bediol^®^, Bedrobinol^®^, Bedrolite^®^ or FM-2 70 or 100 mg/mL	Cannabidiol (CBD), Cannabinol (CBN), Tetrahydrocannabinol (Δ^9^-THC), and Tetrahydrocannabinolic acid (THCA)	No	HPLC-DAD
**Carcieri et al.** [13]	2017	Bediol^®^; Bedrocan^®^; Bedrolite^®^; cannabis olive oil	Cannabinoids (Δ^9^-THC, CBD, THCA, CBDA and CBN)	No	LC-MS
**Trofin et al.** [28]	2012	Cannabis Oil	Cannabinoids (Δ^9^-THC, CBN and CBD)	Long term storage in different conditions; four years in darkness at 4 °C and in laboratory light at 22 °C.	GC–FID, HPLC
**Casiraghi et al.** [11]	2017	Cannabis Olive Oil Preparations	Cannabinoids	No	GC/FID, GC/MS

**Table 2:** Cannabis parameter variation during oil preparation according to the different lipid sources used.

Preparation Step	Bedrocan^®^ Medical Cannabis Based Oil		Bediol^®^ Medical Cannabis Based Oil	
**Extraction solvent**	PhEur grade olive oil (OOPH) *	Medium Chain Triglycerides (MCT) *	PhEur grade olive oil (OOPH) *	Medium Chain Triglycerides (MCT) *
**Oil weight**	50 mL OOPH (d 0.916 = 45.8 g)	50 mL MCT oil (d 0.950 = 47.5 g)	50 mL OOPH (d 0.916 = 45.8 g)	50 mL MCT oil (d 0.950 = 47.5 g)
**Cannabis inflorescence**	5.01 g ± 0.01 Ratio plant/oil (1:10)	5.01 g ± 0.01 Ratio plant/oil (1:10)	5.01 g ± 0.01 Ratio plant/oil (1:10)	5.01 g ± 0.01 Ratio plant/oil (1:10)
**Inflorescence weight after decarboxylation step**	4.63 g ± 0.02 0.37 g ± 0.01 weight loss	4.63 g ± 0.01 0.37 g ± 0.01 weight loss	4.72 g ± 0.02 0.28 g ± 0.01 weight loss	4.75 g± 0.01 0.25 ± 0.01 g weight loss
**Inflorescence and oil weight**	50.43 g ± 0.03	52.12 g ± 0.04	50.52 g ± 0.02	52.24 g ± 0.05
**Macerated oil weight after extraction process**	50.32 g ± 0.02 0.11 g ± 0.01 weight loss	52.00 g ± 0.02 0.12 g ± 0.01 weight loss	50.31 g ± 0.01 0.21 g ± 0.01 weight loss	52.12 g ± 0.01 0.12 g ± 0.01 weight loss
**Macerated oil weight after filtration process**	44.64 g ± 0.02	46.23 g ± 0.03	43.91 g ± 0.02	45.78 g ± 0.02
**Inflorescence weight and oil after filtration process**	5.17 g ± 0.06 inflorescences and oil 0.54 g ± 0.06 oil retained by plant material	5.15 g ± 0.05 inflorescences and oil 0.52 g ± 0.04 oil retained by plant material	5.99 g ± 0.05 inflorescences and oil 1.27 g ± 0.05 oil retained by plant material	5.62 g ± 0.07 inflorescences and oil 0.87 g ± 0.06 oil retained by plant material
**Extraction efficiency**	97.46 %	97.32 %	95.87 %	96.37 %

* = data are expressed as mean (*n* = 3) ± standard deviation.

**Table 3:** Coefficient of variation (CV%) in the concentration of cannabinoids at T0 (ppm) in Bediol^®^ (*n* = 2) and Bedrocan^®^ oils (*n* = 2) between the lipid sources considered.

	Bediol^®^		Bedrocan^®^	
	mean ± SD	CV%	mean ± SD	CV%
CBD	862.5 ± 25.2	2.9	37.5 ± 0.3	1.0
THC	720.2 ± 67.8	9.4	2008.9 ± 102.0	5.1
CBN	5.3 ± 0.04	0.9	8.4 ± 0.7	8.6
CBD-A	96.5 ± 15.5	15.1	30.2 ± 0.1	0.4
THC-A	30.3 ± 0.9	3.0	35.6 ± 2.1	6.0

**Table 4:** Cannabis oil preparation parameters adopted in the present study.

Preparation step	Weight/Duration	Details/Description
Inflorescence weighing	5 g Bedrocan^®^ or Bediol^®^ medical cannabis varieties	Analytical balance—1:10 ratio plant/oil
Inflorescence grinding	2 min	Grinder—Plant material homogenization
Decarboxylation	125 °C; 30 min * * (decarboxylation time is considered when oven reaches the temperature setting)	Laboratory oven without air convection with automatic thermostat - conversion of acid cannabinoids into neutral forms especially for Δ^9^-THC and CBD active compounds
Inflorescence Cooling	10 min; room temperature (25 °C, 60% RH)	
SHAKING	10 min; room temperature (25 °C; 60% RH)	Mechanical rod stirrer—Homogenization of decarboxylated plant material and oil (MCT and olive)
Macerated oil extraction	100 °C; 30 min * * (maceration time is considered when oil reaches the temperature setting)	Magnetic stirrer with heating plate
Oil filtration	5 min	Mechanical press with filter system - separation of plant residues and oil
Magistral oil labelling and storage	Refrigerated storage (4 °C ± 1)	Storage in amber glass containers to prevent photo-oxidation phenomena

## Supplementary Materials

The following are available online, Figure S1: Structural formula of investigated cannabinoids; Table S1: Concentration of cannabinoids (ppm) in Bedrocan oils as a function of the storage times and lipid sources; Table S2. Concentration of cannabinoids (ppm) in Bediol oils as a function of the storage times and lipid sources; Table S3. Concentration of terpenes (ppm) in Bediol oils obtained using OOPH as a function of the storage time; Table S4. Concentration of terpenes (ppm) in Bediol oils obtained using MCT oil as a function of the storage time; Table S5. Concentration of terpenes (ppm) in Bedrocan oils obtained using OOPH as a function of the storage time; Table S6. Concentration of terpenes (ppm) in Bedrocan oils obtained using MCT oil as a function of the storage time.
