# Supplementary material for: Impact of Lipid Sources on Quality Traits of Medical Cannabis-Based Oil Preparations
Source: Molecules. 2020 Jun 30;25(13):2986. doi: 10.3390/molecules25132986 (PMC7412533; doi:10.3390/molecules25132986)
Supplement: Supplementary file 1 [file molecules-25-02986-s001.pdf]

Impact of lipid sources on quality traits of medical cannabis based oil preparations

Alberto Ramella [a], Gabriella Roda[b], Radmila Pavlovic[c]*, Michele Dei Cas[d], Eleonora Casagni[b], Giacomo Mosconi[b], Francisco Cecati[e], Paola Minghetti[b], Carlo Grizzetti[f]

[a] Farmacia Dott.ri Giuliana e Alberto Ramella – SAS. Via A. Diaz 1. Angera (VA). 21021. Italy
[b] Department of Pharmaceutical Sciences. Università degli Studi di Milano. Via L. Mangiagalli 25. Milan. 20133. Italy
[c] Department of Health. Animal Science and Food Safety. University of Milan. Milan. Italy
[d] Department of Health Sciences. Università degli Studi di Milano. Via A.di Rudinì 8. Milan. 20142. Italy
[e] INTEQUI-CONICET. Faculty of Chemistry. Biochemistry and Pharmacy. National University of San Luis. Almirante Brown 1455. CP 5700 San Luis. Argentina
[f] S.S.D. Cure Palliative e Terapia del Dolore. Ospedale di Circolo – Fondazione Macchi. ASST Sette Laghi. Viale L. Borri 57. Varese. 21100. Italy

Supplementary materials

Figure S1. Structural formula of investigated cannabinoids

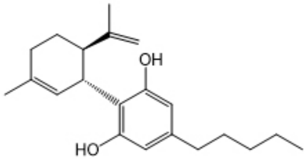

Cannabidiol
CBD

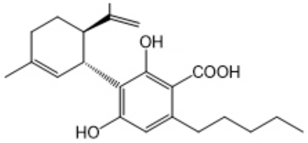

Cannabidiolic acid
CBDA

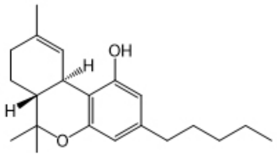

Δ⁹-Tetrahydrocannabinol
Δ⁹-THC

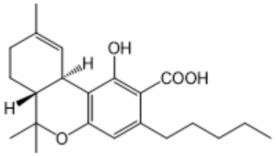

Δ⁹-Tetrahydrocannabinolic acid
Δ⁹-THCA

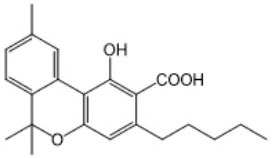

Cannabinol
CBN

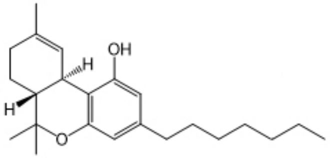

Δ⁹-Tetrahydrocannabiphorol
Δ⁹-THCP

Table S1. Concentration of cannabinoids (ppm) in Bedrocan oils as a function of the storage times and lipid sources

	T 0		T 15		T 30		T 45		T 60		T 75		T 90	
**PhEur grade olive oil (OOPH)**	**mean (n=4)**	**s.d. (±)**	**mean (n=4)**	**s.d. (±)**	**mean (n=4)**	**s.d. (±)**	**mean (n=4)**	**s.d. (±)**	**mean (n=4)**	**s.d. (±)**	**mean (n=4)**	**s.d. (±)**	**mean (n=4)**	**s.d. (±)**
**CBD**	414.18	0.72	361.86	0.46	363.45	10.91	361.59	5.62	154.90	5.19	185.73	1.04	175.81	1.47
**Δ⁹-THC**	23123.96	1082.11	21551.89	293.36	23219.49	1700.18	21469.72	417.86	23160.49	201.16	20777.59	253.82	16425.32	424.88
**CBN**	99.86	10.76	76.79	13.91	157.87	5.82	142.27	12.15	208.94	8.66	276.89	9.21	255.91	2.69
**CBDA**	337.31	0.40	278.82	0.62	295.19	1.98	277.47	0.77	149.05	35.65	143.18	0.99	155.45	0.23
**Δ⁹-THCA**	413.39	0.76	322.85	21.01	358.83	9.39	367.42	2.44	230.84	12.36	266.16	2.42	208.99	39.03
	T 0		T 15		T 30		T 45		T 60		T 75		T 90	
**Medium chain Triglyceride (MCT)**	**mean (n=4)**	**s.d. (±)**	**mean (n=4)**	**s.d. (±)**	**mean (n=4)**	**s.d. (±)**	**mean (n=4)**	**s.d. (±)**	**mean (n=4)**	**s.d. (±)**	**mean (n=4)**	**s.d. (±)**	**mean (n=4)**	**s.d. (±)**
**CBD**	420.13	1.72	361.22	2.29	359.39	0.45	363.17	1.05	156.29	8.69	186.83	1.53	178.99	3.96
**Δ⁹-THC**	21520.15	831.87	22197.35	584.73	20241.89	343.09	21107.45	359.77	22504.11	96.84	20969.17	431.09	17190.08	67.89
**CBN**	88.42	9.78	135.38	8.25	132.12	3.88	128.96	9.69	210.95	5.47	274.80	2.54	250.03	1.36
**CBDA**	335.36	1.36	282.11	0.92	295.75	0.67	278.06	0.47	120.08	0.69	142.87	1.13	156.63	1.22
**Δ⁹-THCA**	379.49	34.07	360.00	9.06	359.09	9.04	385.77	1.59	232.39	6.25	275.69	6.35	245.46	0.78

Table S2. Concentration of cannabinoids (ppm) in Bediol oils as a function of the storage times and lipid sources

	T 0		T15		T 30		T45		T 60		T 75		T 90	
**PhEur grade olive oil (OOPH)**	**mean (n=4)**	**s.d. (±)**	**mean (n=4)**	**s.d. (±)**	**mean (n=4)**	**s.d. (±)**	**mean (n=4)**	**s.d. (±)**	**mean (n=4)**	**s.d. (±)**	**mean (n=4)**	**s.d. (±)**	**mean (n=4)**	**s.d. (±)**
**CBD**	8803.17	174.12	8917.29	263.70	8097.82	374.20	7723.68	242.49	8564.07	31.78	7549.44	122.45	5566.34	133.27
**Δ^9-THC**	7469.21	187.75	7400.85	227.64	7280.60	398.47	6617.54	275.86	7347.22	21.52	6447.71	191.46	5138.76	132.80
**CBN**	59.84	4.11	67.85	2.39	66.94	16.86	65.86	2.26	147.73	1.73	210.70	6.57	200.63	2.04
**CBDA**	651.06	189.29	824.87	28.18	667.30	51.94	707.18	8.84	641.29	30.27	183.98	1.42	479.16	8.89
**Δ^9-THCA**	344.71	34.94	255.91	4.05	258.97	1.78	431.52	6.91	159.62	1.27	185.87	1.43	182.11	1.18
	**T 0**		**T 15**		**T 30**		**T 45**		**T 60**		**T 75**		**T 90**	
**Medium chain Triglyceride (MCT)**	**mean (n=4)**	**s.d. (±)**	**mean (n=4)**	**s.d. (±)**	**mean (n=4)**	**s.d. (±)**	**mean (n=4)**	**s.d. (±)**	**mean (n=4)**	**s.d. (±)**	**mean (n=4)**	**s.d. (±)**	**mean (n=4)**	**s.d. (±)**
**CBD**	8446.55	65.63	8512.23	209.66	7675.90	560.55	8059.71	817.44	8690.26	34.28	7113.56	44.19	4919.21	76.26
**Δ^9-THC**	8534.96	514.43	7545.96	229.91	7294.54	458.91	7628.01	952.03	7809.98	75.19	6362.37	25.09	4759.49	123.04
**CBN**	59.09	2.24	62.10	0.20	63.61	10.57	71.80	13.54	151.27	5.75	198.91	11.76	180.95	21.72
**CBDA**	1195.29	142.96	1370.94	90.16	1097.92	128.50	1195.39	141.57	1120.80	58.21	1054.90	12.10	759.87	25.65
**Δ^9-THCA**	330.30	14.57	302.44	7.08	293.70	8.17	304.02	6.25	193.77	3.71	219.52	7.89	201.35	1.39

Table S3. Concentration of terpenes (ppm) in Bediol oils obtained using OOPH as a function of the storage time

RT	Compound	RI	Storage days													
			T 0		T 15		T 30		T 45		T 60		T 75		T 90	
			Mean	±SD	Mean	±SD	Mean	±SD	Mean	±SD[d]	Mean	±SD[d]	Mean	±SD[d]	Mean	±SD[d]
**Aldehydes**																
8.98	Hexanal	785	n.d.		n.d.		n.d.		n.d.		n.d.		1.32	0.13	0.72	0.01
	tot												***1.32***		***0.72***	
**Alcohols**																
20.01	1-Hexanol	831	5.13	0.36	5.43	1.63	3.28	0.31	3.52	0.97	2.54	3.12	3.21	0.24	2.10	0.03
	tot		***5.13***		***5.43***		***3.28***		***3.52***		***2.54***		***3.21***		***2.10***	
**Esters**																
21.66	Butanoic acid-hexyl ester	1183	5.57	0.63	6.32	1.17	2.37	0.78	2.14	0.24	3.08	3.89	3.15	1.07	1.94	0.09
26.02	Hexanoic acid-hexyl ester	1381	1.01	0.17	0.89	0.30	0.19	0.04	0.29	0.05	0.39	0.48	0.38	0.04	0.31	0.08
29.81	β-Phenethyl acetate	1410	0.68	0.16	0.68	0.11	0.15	0.08	0.21	0.01	0.43	0.50	0.28	0.09	0.14	0.00
	tot		***7.26***		***7.89***		***2.72***		***2.65***		***3.90***		***3.81***		***2.39***	
**Organic acids**																
22.23	Acetic acid	576	n.d.		n.d.		n.d.		2.40	0.20	0.57	0.47	0.31	0.08	0.26	0.03
26.17	Butanoic acid	775	n.d.		n.d.		n.d.		n.d.		n.d.		n.d.		n.d.	
30.32	Hexanoic acid	974	0.69	0.14	0.75	0.48	0.39	0.20	1.39	0.02	2.21	2.70	0.00	0.00	0.00	0.00
32.1	Heptanoic acid	1073	0.68	0.07	0.73	0.18	0.44	0.15	1.43	0.06	1.66	2.06	0.47	0.15	0.22	0.02
33.04	Octanoic acid	1173	0.21	0.07	0.33	0.02	0.11	0.05	0.08	0.00	0.13	0.16	0.04	0.01	0.03	0.00
34.11	Nonanoic acid	1272	0.24	0.08	0.58	0.56	0.31	0.11	0.09	0.01	0.45	0.58	0.13	0.01	0.07	0.03
	tot		***1.81***		***2.39***		***1.25***		***5.38***		***5.02***		***0.94***		***0.57***	
**Monoterpenes**																
9.88	β-Pinene	989	1.56	0.03	1.54	0.36	2.34	0.23	1.28	0.38	0.86	1.03	2.84	0.01	1.36	0.09
12.14	δ-3-Carene	1015	2.41	0.05	2.34	0.02	1.85	0.55	2.22	0.15	1.42	1.63	1.89	0.35	0.95	0.09
12.94	α-Phellandrene	1017	2.57	0.07	3.01	0.17	1.06	0.35	1.13	0.25	1.13	1.34	1.33	0.16	0.87	0.03
13.38	β-Myrcene	1023	115.57	0.70	112.95	2.02	62.15	3.40	38.92	3.77	37.65	42.39	68.51	4.74	54.43	3.17
13.64	α-Terpinene	1026	3.68	0.39	5.63	0.19	1.87	0.16	0.97	0.17	1.25	1.50	2.08	1.05	1.75	1.15
14.48	Limonene	1038	3.92	0.33	5.19	0.56	2.23	0.41	2.63	0.15	2.59	3.03	3.41	1.08	1.83	0.19

14.78	β-Sabinene	1044	7.39	0.13	8.99	1.01	5.20	0.85	4.86	0.61	4.26	4.86	7.86	1.65	4.79	0.54
16.43	γ-Terpinene	1066	4.23	0.25	6.11	0.26	1.93	1.25	1.72	0.20	2.05	2.51	2.32	0.78	2.03	0.48
16.9	β-Ocimene	1070	8.28	2.79	11.75	1.80	5.71	1.37	4.46	0.52	5.44	6.58	6.79	0.97	5.49	0.52
17.26	p-Cymene	1083	22.68	2.97	22.64	5.63	40.70	17.38	66.10	0.65	25.06	28.85	18.30	6.61	5.13	0.42
17.71	α-Terpinolene	1094	58.57	4.55	73.02	11.83	36.00	3.07	31.53	0.31	32.73	40.08	26.55	3.24	19.26	1.05
21.98	Cymenene	1120	13.34	0.32	15.82	4.98	15.78	6.29	18.55	3.57	15.11	18.07	8.37	1.15	4.66	0.75
22.63	4.8-Epoxy-p-menth-1-ene	1177	0.58	0.10	0.50	0.04	0.74	0.18	0.28	0.03	0.28	0.34	0.42	0.04	0.19	0.04
25.33	Fenchyl alcohol	1198	0.37	0.10	0.33	0.14	0.23	0.16	0.15	0.01	0.18	0.22	0.14	0.02	0.13	0.01
25.69	4-Terpineol	1209	n.d.		n.d.		n.d.		n.d.		n.d.		n.d.		n.d.	
27.4	1.8-Menthadien-4-ol	1217	1.86	0.30	1.57	0.32	0.92	0.31	2.00	0.07	1.83	2.25	1.44	0.61	0.66	0.04
27.6	α-Terpineol	1225	2.30	0.91	1.97	0.61	0.97	0.29	2.69	0.01	2.20	2.69	1.55	0.72	1.01	0.31
29.66	trans-2.3-Epoxycaren	1245	0.15	0.02	0.10	0.02	0.06	0.00	0.10	0.02	0.09	0.11	0.05	0.03	0.11	0.06
30.4	P-Cymenol	1293	0.89	0.33	0.77	0.07	0.36	0.01	0.77	0.08	0.59	0.71	0.60	0.17	0.35	0.02
	tot		***250.35***		***274.21***		***180.09***		***180.36***		***134.73***		***154.46***		***105.00***	
**<u>Sesquitepenes</u>**																
25.47	α-Bergamotene	1430	0.48	0.01	0.57	0.14	0.49	0.16	1.20	0.04	0.99	1.24	0.64	0.43	0.29	0.02
25.58	γ-Caryophyllene	1482	26.92	8.00	21.12	8.92	7.57	2.24	21.98	0.17	18.11	22.39	9.17	4.79	5.37	0.19
27.05	α-Humulene	1494	4.95	1.50	3.87	1.85	1.80	0.65	5.52	0.26	4.20	5.26	2.19	1.65	0.89	0.28
27.51	sesquiterpene	1485	n.d.		n.d.		n.d.		n.d.		n.d.		n.d.		n.d.	
28.03	δ-Guaiene	1490	6.56	1.32	4.72	2.17	2.24	0.58	5.23	0.10	4.48	5.53	2.68	1.64	1.01	0.07
28.14	β-Selinene	1507	1.89	0.34	1.36	0.64	0.67	0.22	1.48	0.49	1.51	1.91	0.91	0.59	0.28	0.01
28.33	α-Gurjunene	1519	0.57	0.20	0.59	0.16	0.19	0.09	1.11	0.00	1.30	1.64	0.58	0.17	1.03	0.15
29.19	Selina-3.7(11)-diene	1527	4.99	1.25	3.78	1.99	1.53	0.45	4.20	0.14	3.66	4.51	1.97	1.30	0.44	0.58
	tot		***46.36***		***36.00***		***14.49***		***40.72***		***34.25***		***18.14***		***9.31***	

RT: retention time (min); Mean: Mean value (n = 3); Data are expressed in ppm SD: Standard deviation (n = 3); RI[b]: retention index calculated on a Rtx-Wax (30 m x 0.25 mm x 0.25 m f.t.)

Table S4. Concentration of terpenes (ppm) in Bediol oils obtained using MCT oil as a function of the storage time

RT	Compound	RI	Storage days													
			T0		T15		T30		T45		T60		T75		T90	
			Mean	±SD	Mean	±SD	Mean	±SD	Mean	±SD	Mean	±SD	Mean	±SD	Mean	±SD
**<u>Aldehydes</u>**																
8.98	Hexanal	785	n.d.		n.d.		n.d.		n.d.		n.d.		n.d.		n.d.	
	tot															
**<u>Alcohols</u>**																
20.01	1-Hexanol	831	8.51	1.08	4.24	4.69	6.38	2.50	10.70	4.33	8.80	1.70	12.73	0.09	6.39	1.17
	tot		***8.51***		***4.24***		***6.38***	***2.50***	***10.70***		***8.80***		***12.73***		***6.39***	
**<u>Esters</u>**																
21.66	Butanoic acid-hexyl ester	1183	7.86	0.33	4.13	4.68	3.27	0.53	6.82	1.04	7.89	1.01	5.83	0.81	3.64	1.00
26.02	Hexanoic acid-hexyl ester	1381	1.50	0.31	1.66	0.36	0.34	0.05	0.43	0.16	0.81	0.07	0.88	0.17	0.56	0.27
29.81	β-Phenethyl acetate	1410	0.56	0.06	0.55	0.02	0.12	0.04	0.07	0.04	0.11	0.02	n.d.		n.d.	
	tot		***9.93***		***6.34***		***3.73***		***7.31***		***8.82***		***6.72***		***4.20***	
**<u>Organic acids</u>**																
22.23	Acetic acid	576	n.d.		n.d.		n.d.		n.d.		n.d.		n.d.		n.d.	
26.17	Butanoic acid	775	2.19	0.14	2.24	0.38	0.31	0.11	6.78	0.06	4.87	1.44	0.39	0.03	0.29	0.00
30.32	Hexanoic acid	974	1.36	0.34	1.19	0.05	0.11	0.10	2.08	1.38	2.00	0.44	n.d.		n.d.	
32.1	Heptanoic acid	1073	0.99	0.02	1.04	0.04	0.40	0.08	0.39	0.36	1.64	0.47	0.39	0.07	0.32	0.13
33.04	Octanoic acid	1173	0.68	0.00	0.73	0.03	0.38	0.06	0.32	0.32	0.80	0.07	n.d.		n.d.	
34.11	Nonanoic acid	1272	n.d.		n.d.		n.d.		n.d.		n.d.		n.d.		n.d.	
	tot		***5.23***		***5.20***		***1.20***		***9.57***		***9.31***		***0.78***		***0.61***	
**<u>Monoterpenes</u>**																
9.88	β-Pinene	989	4.52	0.24	5.20	0.34	6.39	1.24	3.71	0.19	3.60	0.26	6.56	0.58	3.17	0.46
12.14	δ-3-Carene	1015	4.86	0.76	5.00	0.37	1.94	0.89	4.03	0.50	5.58	0.50	4.51	2.40	3.38	0.60
12.94	α-Phellandrene	1017	5.39	0.52	5.57	0.08	2.64	0.11	2.80	0.06	4.42	0.39	4.25	0.22	2.31	0.13
13.38	β-Myrcene	1023	186.70	0.42	193.47	16.51	205.80	5.63	114.69	7.62	155.18	25.72	148.95	11.52	132.79	8.57

13.64	α-Terpinene	1026	5.35	1.50	5.61	2.02	3.25	0.09	2.80	0.21	9.93	0.71	5.92	2.02	2.42	0.60
14.48	Limonene	1038	7.17	0.92	7.39	0.34	3.06	0.51	7.12	1.12	8.92	1.03	6.29	1.15	5.42	0.14
14.78	β-Sabinene	1044	15.62	1.36	16.24	2.76	9.55	1.32	11.20	3.76	14.69	0.97	15.00	5.40	12.77	3.98
16.43	γ-Terpinene	1066	6.99	0.52	39.92	46.31	2.88	0.51	4.84	0.76	8.80	0.67	7.05	0.47	5.19	1.49
16.9	β-Ocimene	1070	16.46	0.21	17.07	1.64	10.15	2.50	11.63	0.62	19.90	2.03	18.16	5.66	14.25	1.75
17.26	p-Cymene	1083	25.41	1.98	14.39	16.63	25.98	1.33	52.40	3.33	44.00	6.41	29.35	13.80	11.65	1.01
17.71	α-Terpinolene	1094	101.54	6.85	104.85	1.54	72.55	5.66	76.43	4.70	93.49	0.90	83.02	16.99	48.14	11.28
21.98	Cymenene	1120	17.09	2.64	18.49	0.01	14.67	4.39	38.81	0.64	29.97	3.44	15.93	2.05	6.95	0.59
22.63	4.8-Epoxy-p-menth-1-ene	1177	n.d.		n.d.		n.d.		n.d.		n.d.		n.d.		n.d.	
25.33	Fenchyl alcohol	1198	0.32	0.06	0.37	0.10	0.19	0.01	0.25	0.12	0.24	0.04	0.24	0.08	0.16	0.04
25.69	4-Terpineol	1209	1.43	0.04	1.57	0.04	0.77	0.31	0.84	0.25	0.85	0.09	0.80	0.37	0.69	0.30
27.4	1.8-Menthadien-4-ol	1217	2.57	0.05	2.76	0.13	1.17	0.47	2.60	0.57	3.16	0.25	1.85	0.81	1.41	0.41
27.6	α-Terpineol	1225	3.00	0.82	3.82	0.15	1.16	0.26	3.25	0.52	3.50	0.25	1.88	1.20	1.29	0.53
29.66	*trans*-2.3-Epoxycaren	1245	0.28	0.02	0.31	0.01	0.08	0.00	0.13	0.05	0.22	0.00	n.d.		n.d.	
30.4	P-Cymenol	1293	0.67	0.11	0.80	0.03	0.52	0.19	0.80	0.30	1.02	0.29	0.72	0.32	0.47	0.16
	tot		***405.36***		***442.83***		***362.75***		***338.33***		***407.46***		***350.47***		***252.47***	
**Sesquitepenes**																
25.47	α-Bergamotene	1430	2.78	0.29	3.04	0.32	1.03	0.24	2.00	0.63	2.38	0.62	1.35	0.70	0.69	0.10
25.58	γ-Caryophyllene	1482	39.38	0.56	45.85	3.20	23.62	2.04	35.77	9.44	39.32	6.55	23.16	16.07	16.10	5.50
27.05	α-Humulene	1494	7.90	0.49	9.45	0.58	3.57	1.10	8.55	2.31	9.70	0.91	4.52	3.09	2.69	0.96
27.51	sesquiterpene	1485	0.42	0.15	1.59	0.67	0.41	0.15	1.02	0.34	1.13	0.06	0.79	0.71	0.55	0.26
28.03	δ-Guaiene	1490	4.30	0.09	4.66	0.18	3.48	0.89	7.47	1.40	7.22	0.21	4.14	2.27	1.73	0.31
28.14	β-Selinene	1507	2.67	0.02	2.86	0.11	0.53	0.54	2.81	0.73	3.49	0.65	1.78	1.32	1.08	0.42
28.33	α-Gurjunene	1519	0.60	0.18	0.51	0.02	0.38	0.16	0.89	0.25	0.86	0.16	n.d.		n.d.	
29.19	Selina-3.7(11)-diene	1527	3.11	0.04	3.35	0.13	2.51	0.48	5.43	1.26	6.31	0.64	3.43	2.04	1.68	0.41
	tot		***61.17***		***71.30***		***35.53***		***63.93***		***70.42***		***39.18***		***24.51***	

RT: retention time (min); Mean: Mean value (n = 3); Data are expressed in ppm SD: Standard deviation (n = 3); RI[b]: retention index calculated on a Rtx-Wax (30 m x 0.25 mm x 0.25 m f.t.)

Table S5. Concentration of terpenes (ppm) in Bedrocan oils obtained using OOPH as a function of the storage time

RT	Compound	RI	Storage days													
			T0		T15		T30		T45		T60		T75		T90	
			Mean	±SD[d]	Mean	±SD[d]	Mean	±SD[d]	Mean	±SD[d]	Mean	±SD[d]	Mean	±SD[d]	Mean	±SD
**Aldehydes**																
8.98	Hexanal	785	n.d.		n.d.		n.d.		0.40	0.08	0.60	0.03	1.72	0.17	0.78	0.13
	tot								***0.40***		***0.60***		***1.72***		***0.78***	
**Alcohols**																
20.02	1-Hexanol	831	n.d.		n.d.		n.d.		2.11	0.39	3.38	0.01	3.65	0.37	2.21	0.30
	tot								***2.11***		***3.38***		***3.65***		***2.21***	
**Esters**																
21.66	Butanoic acid-hexyl ester	1183	10.21	0.72	11.37	2.56	4.80	0.18	6.08	1.21	8.01	0.41	4.93	0.47	5.87	1.63
24.93	(-)-Menthyl acetate	1381	n.d.		n.d.		5.11	2.71	n.d.		1.51	0.00	n.d.		1.08	1.53
29.81	β-phenethyl acetate	1410	2.77	0.95	2.23	0.02	0.75	0.08	0.40	0.07	0.54	0.03	0.36	0.04	0.42	0.34
	tot		***12.98***		***13.60***		***10.66***		***6.48***		***10.06***		***5.29***		***7.37***	
**Organic acids**																
22.1	Acetic acid	576	n.d.		n.d.		n.d.		1.03	0.21	0.81	0.04	6.38	0.87	7.01	1.22
26.17	Butanoic acid	775	2.89	0.11	n.d.		n.d.		0.99	0.21	0.37	0.02	n.d.		n.d.	
30.33	Hexanoic acid	974	0.54	0.27	4.28	1.23	2.37	0.34	0.41	0.08	1.33	0.07	0.22	0.02	1.15	1.43
31.96	Heptanoic acid	1073	0.17	0.01	n.d.		n.d.		0.15	0.03	0.36	0.00	0.21	0.02	0.23	0.03
33.04	Octanoic acid	1173	0.63	0.17	1.10	0.42	0.14	0.00	0.07	0.01	0.29	0.00	0.19	0.03	0.20	0.05
34.11	Nonanoic acid	1272	0.56	0.11	0.68	0.11	0.25	0.01	0.15	0.03	0.44	0.00	0.35	0.02	0.32	0.10
	tot		***4.78***		***6.06***		***2.75***		***2.80***		***3.60***		***7.35***		***8.91***	
**Monoterpenes**																
9.88	β-Pinene	989	5.15	0.05	5.62	2.52	9.41	2.91	4.00	0.74	3.06	0.26	4.61	0.44	2.85	0.77
12.14	δ-3-Carene	1015	15.67	1.65	15.31	2.62	16.48	3.68	14.66	2.73	10.37	0.88	12.91	1.24	9.35	0.60
12.94	α-Phellandrene	1017	17.69	1.73	20.98	5.17	6.42	0.21	6.87	1.28	7.43	0.38	6.64	0.64	8.41	0.36
13.38	β-Myrcene	1023	79.03	5.41	84.24	19.46	37.29	2.84	27.33	5.09	39.76	1.83	49.08	4.95	39.27	4.36
13.64	α-Terpinene	1026	22.35	1.80	24.70	6.17	8.57	2.70	8.99	1.67	9.59	0.02	6.55	0.66	9.21	1.53

14.48	α-Linonene	1038	24.28	0.71	26.43	5.80	11.43	1.66	11.50	2.43	12.11	0.62	11.65	1.12	11.91	2.28
14.78	β-Sabinene	1044	34.87	4.96	39.93	12.22	17.45	0.92	18.07	3.36	21.80	1.11	23.54	3.22	19.16	3.61
16.43	γ-Terpinene	1066	18.05	2.01	21.50	5.33	7.14	0.34	9.06	1.69	11.98	0.02	7.34	0.70	10.51	2.03
16.9	β-Ocimene	1070	61.33	0.57	69.03	21.03	30.81	0.42	26.25	4.89	36.62	1.86	32.79	3.15	34.70	2.72
17.26	p-Cymene	1083	53.75	27.96	72.47	29.86	44.99	20.41	40.15	8.83	39.92	2.03	15.16	1.46	25.04	4.22
17.71	α-Terpinolene	1094	340.98	13.36	335.47	87.70	171.57	4.28	147.70	27.49	198.41	10.08	216.78	56.06	195.03	7.82
21.98	Cymenene	1120	20.30	0.81	26.88	9.10	21.41	4.98	30.03	5.59	29.19	1.48	8.43	0.81	11.57	2.78
22.63	4.8-epoxy-p-Menth-1-ene	1177	2.00	0.40	2.42	0.28	34.23	17.58	1.44	0.27	2.32	0.00	1.68	0.17	1.62	0.65
23.13	cis-Menthone	1198	n.d.		n.d.		10.98	3.42	n.d.		0.86	0.04	0.55	0.62	0.42	0.13
24.66	Linalool	1209	5.41	1.00	5.48	0.09	2.92	0.42	6.27	1.01	3.66	0.19	1.92	1.31	2.69	3.15
26.53	Menthol	1217	0.00	0.00	0.00	0.00	13.67	9.27	0.55	0.10	9.80	0.02	n.d.		n.d.	
27.41	1.8-Menthadien-4-ol	1225	2.12	0.07	2.59	0.10	0.00	0.00	1.91	0.35	1.07	0.00	0.95	0.09	1.99	0.87
27.6	α-Terpineol	1245	7.94	0.16	10.58	1.99	5.66	0.47	7.55	1.41	6.34	0.01	4.49	0.45	5.13	3.07
29.66	*trans*-2.3-Epoxycaren	1293	0.28	0.01	9.24	2.55	0.00	0.00	0.16	0.03	0.00	0.00	0.18	0.02	0.39	0.25
30.4	p-Cymenol		3.10	0.24	0.34	0.10	0.59	0.24	2.40	0.45	2.63	0.17	1.49	0.20	2.48	1.48
	tot		***714.30***		***773.22***		***451.03***		***364.90***		***446.93***		***406.73***		***391.72***	
**Sesquitepenes**																
25.58	γ-Caryophyllene	1482	36.90	6.05	40.26	3.45	29.14	4.84	38.82	7.23	5.14	0.26	13.11	1.32	23.12	11.8
27.05	α-Humulene	1494	8.37	0.77	8.65	0.10	6.83	0.44	9.41	1.75	1.69	0.09	3.26	0.45	5.36	2.96
28.03	δ-Guaiene	1485	8.25	0.13	8.48	1.06	6.33	0.57	6.66	1.58	7.78	0.40	2.34	0.22	3.50	1.75
28.14	β-Selinene	1490	2.29	0.24	2.55	1.09	1.77	0.29	2.41	0.45	2.73	0.01	0.80	0.05	1.44	0.38
28.33	α-Gurjunene	1507	2.04	0.13	2.46	0.53	1.48	0.03	2.30	0.43	2.43	0.12	0.75	0.07	1.77	0.36
29.02	β-Maaliene	1519	1.30	0.06	1.31	0.05	0.48	0.67	1.04	0.19	1.35	0.00	3.46	0.20	1.27	0.46
29.19	Selina-3.7(11)-diene	1527	15.30	0.20	8.88	9.15	10.21	1.52	12.58	2.34	13.88	0.91	4.64	0.63	7.96	3.23
	tot		***74.44***		***72.59***		***56.24***		***73.22***		***35.00***		***28.36***		***44.42***	

RT: retention time (min); Mean: Mean value (n = 3); Data are expressed in ppm SD: Standard deviation (n = 3); RI^b: retention index calculated on a Rtx-Wax (30 m x 0.25 mm x 0.25 m f.t.)

Table S6. Concentration of terpenes (ppm) in Bedrocan oils obtained using MCT oil as a function of the storage time

RT	Compound	RI	Storage days														
			T0		T15		T30		T45		T60		T75		T90	
			Mean	±SD	Mean	±SD	Mean	±SD	Mean	±SD	Mean[c]	±SD	Mean[c]	±SD[d]	Mean[c]	±SD[d]
**Aldehydes**																
8.98	Hexanal	785	n.d.		n.d.		n.d.		n.d.		n.d.		n.d.		n.d.	
	**tot**															
**Alcohols**																
20.02	1-Hexanol	831	n.d.		n.d.		n.d.		4.95	0.13	4.89	0.04	4.10	0.05	4.61	0.15
	tot								***4.95***		***4.89***		***4.10***		***4.61***	
**Esters**																
21.66	Butanoic acid-hexyl ester	1183	11.57	3.73	12.37	1.94	7.75	0.45	5.68	0.14	15.78	0.14	11.63	1.50	5.96	0.44
24.93	(-)-Menthyl acetate	1381	n.d.		n.d.		8.21	4.22	n.d.		n.d.		n.d.		n.d.	
29.81	β-phenethyl acetate	1410	2.98	0.27	2.54	0.98	1.21	0.11	n.d.		n.d.		n.d.		n.d.	
	tot		***14.55***		***14.90***		***17.17***		***5.68***		***15.78***		***11.63***		***5.96***	
**Organic acids**																
22.1	Acetic acid	576	n.d.		n.d.		n.d.		10.72	0.27	3.97	0.04	16.78	0.22	12.54	4.71
26.17	Butanoic acid	775	3.23	0.70	n.d.		0.95	1.35	9.97	0.25	3.56	0.74	0.90	0.01	0.47	0.03
30.33	Hexanoic acid	974	0.56	0.15	4.60	0.42	3.81	0.47	0.39	0.01	0.37	0.00	0.30	0.12	0.11	0.01
31.96	Heptanoic acid	1073	0.19	0.06	n.d.		n.d.		0.12	0.00	0.19	0.00	0.15	0.00	0.13	0.00
33.04	Octanoic acid	1173	0.68	0.02	1.34	0.95	0.22	0.01	0.60	0.02	0.35	0.00	0.38	0.00	0.13	0.00
34.11	Nonanoic acid	1272	0.62	0.03	0.80	0.42	0.40	0.02	0.31	0.01	0.37	0.00	0.17	0.02	0.28	0.01
	tot		***5.28***		***6.74***		***5.38***		***22.12***		***8.82***		***18.67***	***0.38***	***13.66***	
**Monoterpenes**																
9.88	β-Pinene	989	6.63	0.34	5.84	0.47	15.16	4.41	6.15	0.16	5.87	0.05	9.58	2.54	8.78	0.28
12.14	δ-3-Carene	1015	17.37	2.62	16.82	3.54	26.56	5.43	25.40	0.64	25.00	1.20	22.32	5.91	16.78	1.25
12.94	α-Phellandrene	1017	19.62	3.10	22.71	3.05	10.36	0.14	10.54	0.27	26.69	0.24	12.34	3.27	9.24	2.58

13.38	β-Myrcene	1023	88.01	16.48	91.49	13.75	60.20	3.42	57.63	1.46	85.61	0.78	73.38	0.94	59.21	1.87
13.64	α-Terpinene	1026	25.36	8.40	26.73	3.50	13.80	4.10	16.92	0.43	20.56	0.19	18.82	0.24	15.58	1.16
14.48	α-Linonene	1038	27.17	6.14	28.77	4.66	18.44	2.32	16.56	0.42	30.40	0.28	18.89	5.00	21.02	5.88
14.78	β-Sabinene	1044	38.46	4.38	42.73	3.12	28.20	2.02	29.60	0.75	41.12	0.38	39.73	0.51	30.39	0.96
16.43	γ-Terpinene	1066	20.57	7.41	23.27	3.10	11.55	0.78	14.10	0.36	23.34	0.21	20.71	2.66	16.45	1.22
16.9	β-Ocimene	1070	68.82	16.86	73.89	5.50	49.78	1.64	47.02	2.83	78.10	0.71	58.64	7.55	56.30	4.18
17.26	p-Cymene	1083	64.38	46.75	75.92	3.07	72.98	34.36	70.81	79.87	11.18	0.10	74.46	9.58	38.01	10.63
17.71	α-Terpinolene	1094	381.13	82.33	389.63	35.15	277.07	1.57	241.02	6.10	344.83	3.15	339.90	4.37	323.25	10.19
21.98	Cymenene	1120	22.92	6.70	28.57	1.11	34.66	8.71	57.87	1.46	60.50	0.55	26.01	0.33	31.68	2.35
22.63	4.8-epoxy-p-Menth-1-ene	1177	2.19	0.12	2.69	0.71	55.02	27.33	0.00	0.00	3.21	0.03	2.28	0.03	n.d.	
23.13	cis-Menthone	1198	n.d.		n.d.		17.68	5.19	n.d.		1.92	0.02	0.58	0.01	0.53	0.01
24.66	Linalool	1209	5.93	0.43	6.20	2.22	4.71	0.59	3.72	0.09	6.15	0.06	4.34	0.06	3.06	0.04
26.53	Menthol	1217	n.d.		n.d.		21.94	14.54	n.d.		0.92	0.01	n.d.		1.64	0.12
27.41	1.8-Menthadien-4-ol	1225	2.39	0.68	2.92	0.98	n.d.		2.61	0.07	3.97	0.16	2.05	0.03	1.67	0.02
27.6	α-Terpineol	1245	8.90	2.09	11.59	2.24	9.13	0.58	9.68	0.25	11.94	0.11	8.59	0.11	4.99	0.16
29.66	*trans*-2.3-Epoxycaren	1293	n.d.		n.d.		n.d.		n.d.		n.d.		n.d.		n.d.	
30.4	p-Cymenol		3.45	0.61	0.37	0.03	0.96	0.41	12.63	14.31	3.86	0.04	2.73	0.04	1.91	0.06
	tot		***803.31***		***850.14***		***728.20***		***622.27***		***785.16***		***735.34***		***640.48***	
**<u>Sesquitepenes</u>**																
25.58	γ-Caryophyllene	1482	40.59	3.74	44.97	13.21	47.00	6.91	54.82	1.39	73.98	0.68	49.61	0.64	25.95	0.82
27.05	α-Humulene	1494	9.29	1.52	9.80	3.57	11.03	0.50	n.d.		20.46	0.19	8.32	1.07	4.61	0.07
28.03	δ-Guaiene	1485	9.24	2.20	9.40	2.40	10.21	0.73	12.55	0.32	16.46	0.15	7.50	0.97	2.52	0.19
28.14	β-Selinene	1490	2.60	0.93	2.66	0.15	2.86	0.41	3.87	0.10	5.69	0.12	3.19	0.04	1.27	0.02
28.33	α-Gurjunene	1507	2.28	0.44	2.68	0.44	2.40	0.09	3.38	0.09	5.04	0.05	2.59	0.33	1.04	0.01
29.02	β-Maaliene	1519	1.47	0.44	1.47	0.50	0.76	1.07	1.73	0.04	2.44	0.02	13.73	0.18	0.52	0.04
29.19	Selina-3.7(11)-diene	1527	17.21	4.59	12.03	14.17	16.47	2.14	22.22	0.07	28.50	0.26	16.30	2.10	7.69	0.24
	tot		***82.69***		***83.01***		***90.72***		***98.56***		***152.56***		***101.24***		***43.60***	

RT: retention time (min); Mean: Mean value (n = 3); Data are expressed in ppm SD: Standard deviation (n = 3); RI[b]: retention index calculated on a Rtx-Wax (30 m x 0.25 mm x 0.25 mm f.t.)
